# Environmental barriers meet functional limitations: an exploratory qualitative study on the usage and experience of outdoor gym among older adults

**DOI:** 10.3389/fpubh.2026.1861748

**Published:** 2026-07-15

**Authors:** Tong-Yue Zhou, Hong-Jian Lu, Caimin Li, Hui Lyu

**Affiliations:** School of Architecture and Planning, Foshan University, Foshan, Guangdong, China

**Keywords:** older adults, outdoor fitness equipment, outdoor gym, public health, urban park

## Abstract

**Introduction:**

Outdoor gyms are widely used in urban public spaces, yet there is a lack of specialized research for older adults in urban parks from the perspective of functional limitation.

**Methods:**

This study conducted in-depth interviews with 30 older adults via purposive sampling and used thematic analysis to explore their usage and experience of outdoor gym in urban park and related environmental and individual influencing factors.

**Results:**

Results show that outdoor gym have positive impacts on the physical, psychological and social health of older adults. The main motivations include pursuing physical health and satisfying social and emotional needs. Individual constraints involve declines in lower limb function, balance, flexibility, as well as visual and cognitive impairments. Environmental support factors include pleasant natural settings, low noise, comfortable thermal conditions and diverse equipment, while environmental pressure factors include bad weather, insufficient or poorly maintained equipment, noise and inadequate lighting. Older adults proposed optimization suggestions covering safety, information accessibility, comfort and equipment suitability.

**Conclusion:**

This study deepens the understanding of environment-behavior interactions in older adults with functional limitations, offering a valuable empirical baseline for rehabilitation-oriented design for healthy aging.

## 1 Introduction

Over the last few years, the installation of outdoor gym, or fitness zone, outdoor fitness equipment, has surged in public parks globally due to its low cost, practicality, enjoyable and accessibility (1–4). These spaces cater to all age groups by supporting a wide range of physical activities, from aerobic and strength training to flexibility and balance exercises. Moreover, research shows that “green exercise” provides greater physical and mental health benefits than exercising alone (5). Therefore, by combining physical activity with nature, outdoor gyms may have a clear advantage over indoor facilities in promoting public health. However, the spatial design of outdoor gym often lacks dedicated and professional guidelines, leading to inefficient resource utilization and even potential safety risks (6). To address the severe challenge of population aging, the World Health Organization (WHO) set healthy aging as a strategic goal: the process of developing and maintaining functional ability to enable well-being in older age (7). Physical inactivity poses a major health challenge, particularly for older adults. Physical environmental factors are well documented as critical influences on their physical activity (8–10). The World Health Organization (WHO) Age-Friendly Cities framework provides a globally recognized standard for designing urban environments that support healthy ageing. Among its eight core domains, outdoor spaces and buildings is a fundamental dimension that emphasizes the provision of safe, accessible, and well-equipped outdoor public spaces for older adults (11). Outdoor gyms in urban parks, as key components of age-friendly outdoor spaces, directly influence older adults’ physical activity participation and daily exercise experiences. Developing age-friendly outdoor gym and promoting scientific, routine physical activity for older adults are key pathways to achieving healthy aging.

Over the past decade, interdisciplinary scholars have accumulated a growing body of research on outdoor gym, which has been synthesized in several review articles (12–14). These publications collectively compartmentalize existing literature into three core thematic dimensions. First, research on outdoor gym usage patterns has included surveys of user characteristics, temporal trends, usage behaviors, and motivations (12, 15). Second, for health benefits, sports science and medicine scholars have recently investigated exercise prescription effectiveness, focusing on outdoor gym-based interventions’ health impacts on specific populations (2, 16, 17). Third, regarding environment design, studies have identified microclimate, spatial location, planar form, side interface morphology, equipment layout, and seating as important environmental factors affecting usage rates, highlighting the critical role of environment in shaping equipment fitness behaviors (18, 19).

However, extant research on outdoor gym predominantly focused on all-age populations, with limited attention to older adults’ special needs and the impact of individual factors on their equipment fitness behavior. Previous research on the age-friendly outdoor gym has focused primarily on fitness equipment itself (20, 21) with limited attention to the spatial environment. Growing experimental evidence indicates that fitness interventions using outdoor gym facilities can improve physical function, reduce fall risks and enhance quality of life among older adults (16, 22). In recent years, some scholars have explored older adults’ usage experiences with outdoor gym, initially identifying their usage patterns, motivations, perceived benefits, environmental factors and their suggestions (23–25). Notably, environmental requirements within the aging population vary depending on individual physical capacities. While qualitative research confirms that general health status acts as a pivotal usage barrier (26), quantitative evidence shows that mobility-limited individuals have a significantly higher demand for nearby benches and resting infrastructure than those without such limitations (27).

Outdoor gyms play a pivotal role in enabling outdoor rehabilitation for older adults with functional limitations. Lee and Ho reviewed existing evidence and proposed that age-friendly outdoor gyms should target not only preventive exercise but also rehabilitative activities (28). Consistently, a recent participatory study shows outdoor gym could serve as outdoor rehabilitation ground, including walking and fall prevention training (26). Older adults with age- or illness-induced functional impairments have unique spatial design demands for outdoor gym, such as ample wheelchair access between fitness equipment and sufficient handrail facilities (29). Concurrently, an emerging body of literature indicates that well-designed public green spaces can transition from mere recreational spots to outdoor rehabilitation training grounds, significantly improving the balance, motor recovery, and psychological well-being of older adults (16, 30, 31).

Nonetheless, current literature typically treats these physical constraints as a homogenous category, failing to granularly address specific functional declines—such as gait instability, lower limb limitations, and visual or cognitive deficits. Since distinct impairments uniquely alter spatial perception and apparatus manipulation, this study adopts a dedicated “functional limitation” perspective to comprehensively deconstruct older adults’ actual experiences and usage within outdoor gyms.

The ecological model of aging clarifies the correlations among individuals, environments and behaviors, which provides an theoretical foundation for this research. This model holds that individual behaviors as the outcome of the transaction between individual competence and environmental demands (32). Notably, the engagement in equipment-based exercise is the result of the interaction between the environment and the individual. Aging is often accompanied by varying degrees of decline in mental, sensory, and motor functions (33). Functional limitation is a critical factor limiting older adults from participating in equipment fitness activities; thus, it is becoming increasingly urgent to create outdoor activity-friendly environments for older adults with functional limitations in aging society. To the best of our knowledge, no research has explored the impact of various types of functional limitations on equipment fitness behavior, nor their specific requirements for the environment.

Given the socioeconomic gap between China and developed countries, research and design on the age-friendly outdoor gym in urban park remain in the initial stage. Driven by the “Healthy China 2030” initiative, fitness equipment has been mass-installed nationwide, yet its homogenous design lacks design guidelines for older adults with functional limitations. Within high-density urban environments, outdoor gym in urban park represent rare outdoor rehabilitation grounds for this vulnerable group, whose specific needs are often overlooked. Additionally, the unique collective social atmosphere among Chinese older adults creates a double-edged sword, intertwining emotional support with potential risks to self-esteem during exercise. This distinct ecology, woven from administrative execution, spatial crowding, and collective socialization, urgently requires localized empirical evidence to build a reliable evidence base for localized age-friendly renovations. Therefore, this study aims to (1) explore the current usage patterns of urban park outdoor gyms among older adults with functional limitations, (2) clarify the specific individual and environmental factors influencing this usage, and (3) understand the distinct requirements of this vulnerable population within outdoor gym. This research expands the research on outdoor gyms by promoting refined and age-friendly design in urban parks from the perspective of functional limitations. Ultimately, these findings offer an evidence-based foundation for subsequent academic research and design practices.

## 2 Research methods

### 2.1 Study design

This study adopted a descriptive qualitative design underpinned by naturalistic research principles (34). Such research design lays emphasis on depicting participants’ authentic lived experiences within real-life settings, reduces subjective interpretation from researchers and focuses on presenting research phenomena as they naturally occur. Qualitative research methods are highly valuable for exploring cutting-edge fields, especially when greater data richness and depth are required. Based on the ecological model of aging, the researchers employed a thorough, in-depth reading of the participants’ accounts that allowed them to gain a comprehensive understanding of their usage and experience of outdoor gym in urban park. Qualitative Research (COREQ) guidelines for qualitative research reporting (35).

### 2.2 Data collection

Aligned with the study objectives, we developed a structured interview guide that was iteratively refined across multiple rounds of deliberation to optimize its structure, content, and wording. A survey was first conducted to collect information regarding the demographic and health characteristics of the older adults, including their age, sex, diseases, and functional limitations. The main questions were as follows: (1) Could you share your feelings about using outdoor gyms, including what benefits you gain, why you choose to exercise here and how you usually use these facilities? (2) Can you describe how your functional limitations affect your outdoor activities? (3) What barriers and facilitators have you encountered when using the outdoor gym? and (4) How do you hope to improve the outdoor gym? (The complete interview guide is available in the Appendix).

This study adopted purposive sampling rather than pure convenience sampling for participant recruitment. Researchers conducted preliminary field observations at the outdoor gyms to identify older adults who appeared to have functional limitations by examining their movement performance when using fitness facilities, and then invited them to participate in the study. Prior to the formal interview, a demographic and health status questionnaire was used to further confirm participants’ eligibility. All interviews were conducted between August and December 2024 in Guangzhou, covering late summer, autumn and early winter. During this period, the average daily outdoor temperature ranged from 15 °C to 32 °C. All data collection was completed during daytime hours (8:00–18:00) at the selected urban park outdoor gyms. Each participant’s interview duration was 20–40 min. The length of the interviews was controlled by the participants’ ability to participate after somewhat strenuous exercise and what they wanted to talk about. The interviews were conducted by the first author and the second author, who have research experience in environmental gerontology and qualitative methods. The researchers had no prior relationship with participants and remained reflexive throughout data collection to minimize personal bias. Each interview was first transcribed verbatim from field notes and audio recordings by one interviewer, then cross-checked by a second, and finally forwarded to the research team for data collation, coding, and analysis.

### 2.3 Participants and settings

**Figure 1:**
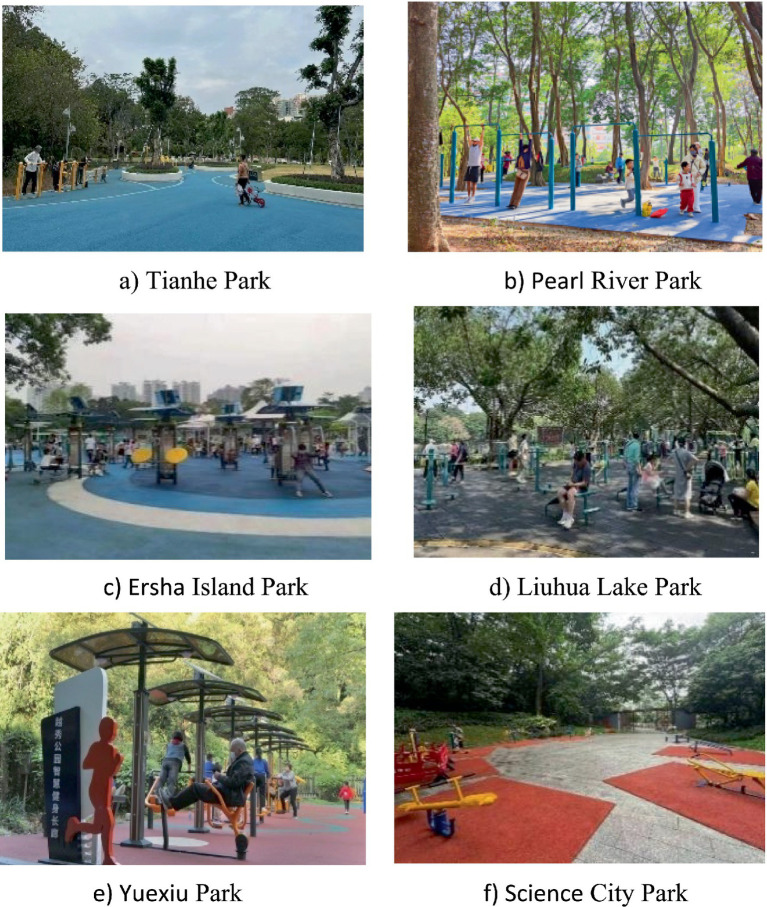
Outdoor gyms in the six urban parks. **(a)** Tianhe Park; **(b)** Pearl River Park; **(c)** Ersha Island Park; **(d)** Liuhua Lake Park; **(e)** Yuexiu Park; **(f)** Science City Park.

The participant recruitment criteria were as follows: (1) Aged 60 years or older; (2) With at least one type of functional limitation; (3) Capable of completing fitness activities independently or with assistive device support; (4) Utilizing park fitness facilities at least once weekly; (5) Able to clearly articulate personal experiences and needs in Mandarin or local Cantonese, with no severe cognitive or language impairments that would prevent participation in an interview, as verified through brief verbal communication during recruitment. To ensure spatial heterogeneity of the sample, six outdoor gym in urban park were chosen across three types of areas in Guangzhou (Figure 1): central urban districts (Tianhe Park, Yuexiu Park, Ersha Island Park), emerging development zones (Science City Park), and waterfront areas (Liuhua Lake Park, Pearl River Park). Before the commencement of the study, ethical approval was obtained from the Human Research Ethics Committee (HREC) of The University of Foshan (Reference number: FUME2024015).

During recruitment, interviewers were required to purposively ensure participant diversity by observing demographic and functional characteristics: gender, age, and walking assistive devices. Thirty older adults (18 males, 12 females), aged 60–80 years, voluntarily participated in the interviews. Qualitative exploratory research generally adopts a widely recognized sample size range of 15 to 30 participants (36). Recruitment was terminated upon achieving data saturation, and 30 participants were ultimately enrolled to ensure adequate data richness. Based on self-reports, participants primarily presented with chronic conditions (hypertension, osteoarthritis, diabetes, cardiovascular disease) and functional impairments (visual decline, cognitive decline, lower extremity dysfunction, poor cardiopulmonary endurance, negative emotional states) (Table 1).

**Table 1:** Health characteristics of the 30 participants.

Characteristic	Number and proportion
Age	60–70 years (*n* = 21, 70.0%); 71–80 years (*n* = 9, 30.0%)
Gender	Male (*n* = 18, 60.0%); Female (*n* = 12, 40.0%)
Condition	Osteoarthritis (*n* = 5, 16.7%); Diabetes (*n* = 3, 10.0%); Hypertension (*n* = 3, 10%); Osteoporosis (*n* = 2, 6.7%); Heart disease (*n* = 1, 3.3%)
Functional limitation	Visual decline (*n* = 10, 33.3%); Cognitive decline (*n* = 8, 26.7%); Lower extremity dysfunction (*n* = 6, 20.0%); Poor cardiopulmonary endurance (*n* = 6, 20.0%); Negative emotional states (*n* = 6, 20.0%); Auditory decline (*n* = 4, 13.3%); Spinal dysfunction (*n* = 4, 13.3%); Poor balance (*n* = 3, 10.0%); Impaired fine motor skills (*n* = 3, 10.0%); Upper extremity dysfunction (*n* = 2, 6.7%); Poor muscular endurance (*n* = 2, 6.7%); Poor physical coordination (*n* = 2, 6.7%); Respiratory decline (*n* = 1, 3.3%); Difficulty with head movement (*n* = 1, 3.3%); Decreased tactile sensation (*n* = 1, 3.3%)
Self-rated health status	Good (*n* = 16, 53.3%); Very good (*n* = 8, 26.7%); Fair (*n* = 6, 20.0%)

### 2.4 Data analysis

The researchers transcribed the audio recordings of the interviews verbatim and used thematic analysis to analyze the data (37). The procedures for thematic analysis were as follows: (1) the primary coder (1st author) familiarized with the data, and then the researchers (2) generated the initial codes, (3) searched the themes, (4) reviewed the themes, (5) defined and named the themes, and (6) produced the report. The researchers used explanatory analysis, a combination approach of induction and deduction, to analyze the data (38). Themes and subthemes related to influencing factors of participants in using the outdoor gym were analyzed deductively using the ecological model of aging framework (32), while themes and subthemes related to their usage and suggestions on outdoor gym were analyzed inductively. NVIVO 12 was used for coding. Verbatim excerpts from older adults were used to substantiate corresponding themes.

### 2.5 Rigour

To ensure the study’s rigour, Lincoln and Guba’s (39) four criteria (credibility, transferability, dependability, confirmability) were applied. Regarding credibility, the research team engaged in prolonged data immersion and repeated reading of all transcripts to interpret participants’ experiences accurately. Two researchers independently coded the dataset and resolved discrepancies through discussion and expert consultation. Data collection was concluded once data saturation was achieved to guarantee reliable information. For transferability, detailed descriptions of participants and research contexts were provided to support judgements on the generalizability of findings. The research team maintained a complete audit trail of recordings, transcripts, and coding records, and developed a standardized codebook to ensure dependability. All findings were derived directly from raw data to minimize subjective bias and ensure confirmability.

## 3 Research findings

The analysis led to the development of the following four themes: (1) Current Usage; (2) Individual influencing factors; (3) Environmental influencing factors; (4) Optimization Recommendations each theme had two to four sub-themes, which, together with their units of meaning, allowed us to explore participants’ usage and experience of outdoor gyms (Table 2).

**Table 2:** Units of meaning explaining participants’ usage and experience of outdoor gym.

Theme	Subtheme	Units of meaning
1. Current usage	1.1 Perceived Health Benefits	Physical functions, mental well-being, social health
	1.2 Usage Motivation	Maintaining physical health, fulfilling emotional needs
	1.3 Usage patterns	Exercise duration, exercise time slot, exercise frequency, frequently used equipment
2. Individual influencing factors	2.1 Lower limb function	equipment choice, pavement condition, exercise intensity
	2.2 Balance and flexibility	equipment choice
	2.3 Visual impairment	understand how to use fitness facilities, adjust fitness equipment
	2.4 Cognitive decline	understand how to use fitness facilities
3. Environmental influencing factors	3.1 Environmental support factors	favorable natural environment, quiet and low-interference soundscape, thermally comfortable microclimate, equipment diversity and usability
	3.2 Environmental press factors	adverse weather conditions, inadequate equipment, poor equipment maintenance, noise disturbance, inadequate lighting
4. Optimization recommendations	4.1 Safety Assurance	pavement condition, equipment maintenance, equipment arrangement
	4.2 Information Accessibility	signage, instructions, and safety labels, exercise guidelines
	4.3 Physical and Mental Comfort	shade structures and rain shelters, comfortable rest areas, storage, and drinking water
	4.4 Appropriateness of Fitness Equipment	the variety and quantity of equipment, low-intensity equipment, professional support for safe exercise

### 3.1 Current usage

#### 3.1.1 Perceived health benefits

Outdoor gyms exert positive health influences on older adults by promoting physical performance and psychosocial well-being. Physically, participants reported that exercising on outdoor fitness equipment enhanced key physical functions, including joint flexibility, muscle strength and cardiorespiratory fitness, while also contributing to better glycemic control, blood pressure regulation, weight management, sleep quality and chronic disease prevention. Compared with other exercise modalities, equipment-based training delivers more targeted physiological benefits. “*I still take daily walks, but equipment-based training is more systematic, enabling targeted conditioning of specific muscle groups*”(N11). Psychologically, participants reported that outdoor fitness equipment use helped regulate mood, relieve stress, and enhance mental well-being. “*These workouts keep me energized and make me feel I’m not aging too quickly*” (N7). Socially, participants reported that outdoor fitness equipment use fostered social interaction and enriched their quality of life. These health benefits appear to act synergistically, with one older adult participant noting, “*I find this fitness equipment effective for strengthening my muscles and joints. Working out boosts my mood, eases my anxiety, and helps me connect with many like-minded friends here*” (N7).

#### 3.1.2 Usage motivation

Gyms maintaining physical health is the primary goal cited by most older adults using urban park fitness trails. The use of fitness equipment functions as a non-pharmacological intervention for disease rehabilitation. Many participants reported following their physicians’ advice to maintain health via outdoor fitness equipment. For instance, one participant noted, *“My doctor also recommended I use fitness equipment more regularly, as it helps alleviate osteoporosis”* (N29). Beyond promoting physical health, outdoor gym also serve as vital venues for older adults to restructure daily routines and combat social isolation following significant life-role transitions. Many participants reported feeling bored at home post-retirement; at outdoor gym, they can not only exercise but also make friends, socialize, and alleviate loneliness. For instance, one participant commented, “*After retirement, I felt bored and cooped up at home. Coming here allows me to not only exercise but also make friends*” (N5).

#### 3.1.3 Usage patterns

The majority of respondents used fitness equipment for 30 min to one hour, while a tiny minority exercised for 2–3 h. Most respondents exercised with outdoor fitness equipment during the daytime, particularly in the morning. Most respondents were frequent users of outdoor gym, with 80% using the facilities more than three times weekly and over 20% reporting daily use. Strength-based and aerobic fitness equipment were the two most commonly used types among participants with rowing machines (strength-based), leg press machines (strength-based), and elliptical trainers (aerobic) the most popular. In addition, participants demonstrated extensive demand for three other equipment categories: massage devices (e.g., leg and back massagers), coordination-focused equipment (e.g., waist twisters, Tai Chi pushers, shoulder rotators), and flexibility-enhancing apparatus (e.g., lumbar and back stretch frames).

### 3.2 Individual influencing factors

Lower limb function influences the use of outdoor gym, including equipment choice; for instance, one participant commented, “*My knee problems have affected my use of standing equipment such as elliptical trainers*” (N2).” *Knee joint pain made it difficult to complete the full range of motion when using the leg press machine*” (N7). Participants with lower limb dysfunction have higher requirements for pavement condition; to mitigate risks, they may actively adjust their exercise routes—avoiding tile-paved areas and opting for rubber-matted zones. One participant described, “*After rain, the ground was as slippery as oil, so I could only move slowly*” (N20). The resistance of fitness equipment may exceed older adults’ tolerance range; for instance, arthritis patients require low-impact exercise.” *The park rowing machine has excessive resistance; using it sometimes causes strain and sharp pains. I have joint pain issues, so I take special care with my exercise methods to protect my joints*” (N20). Neuropathy from chronic diseases in older adults impairs plantar tactile sensation and proprioception, heightening their need for exercise intensity feedback. One diabetic patient stated, “*I do not even notice blisters forming on my soles when the treadmill speed is slightly fast. My diabetes does not directly affect my use of fitness equipment, but I pay closer attention to controlling exercise intensity”* (N30).

With advancing age, older adults experience a decline in balance, which restricts their use of fitness equipment. Unprotected devices such as balance beams are rarely utilized by any respondents. Similarly, reduced flexibility further influences the selection of fitness equipment among older adults. “*Joint stiffness makes it difficult for me to use equipment requiring a wide range of motion*” (N8). “*Spinal stiffness does affect my use of fitness equipment requiring bending movements, such as stretching machines*” (N9).

Visual impairments hinder participants’ ability to understand how to use fitness facilities. This is attributable to equipment operational complexity: traditional fitness apparatus lack usage instructions or have overly small text, while smart devices have undersized touchscreen fonts and no voice prompts—both creating significant operational barriers for visually impaired seniors. “*Most fitness equipment lacks usage instructions; when available, the text is too small for us to read clearly*” (N4). “*My eyesight is poor, so I sometimes cannot read fitness equipment instructions clearly and have to rely on others or figure out how to use them on my own*” (N12) “*I cannot clearly read the instructions and guidelines on fitness equipment. Larger fonts or audio instructions, I believe, would let me use them more safely and confidently*” (N12) Furthermore, some fitness equipment requires adjustment to individual height and physical capabilities, posing a significant challenge for visually impaired participants. “*Take the back stretcher as an example—I cannot clearly read its scales and instructions, so I often need assistance adjusting it to the proper position*” (N5). Cognitive decline also hinders participants’ ability to use fitness equipment: for instance, one participant commented, “*I believe more prompts and guidance are needed, such as installing instruction boards next to the equipment*” (N4).

### 3.3 Environmental influencing factors

#### 3.3.1 Facilitating environmental support factors

Favorable natural environment: participants viewed fresh air and adequate greenery along outdoor gym as paramount, recognizing them as key environmental facilitators of physical activity. Many participants reported that greenery and fresh air promoted relaxation during their visits. Several participants emphasized the restorative benefits of water features (Park B and D), noting that the cooler microclimate and soothing sounds enhanced their exercise experience. As one participant described it: *“Being by the lake provides a cooling sensation; exercising to the sound of water makes time pass unnoticed, and the scenery significantly enhances my overall comfort”* (N6). Quiet and low-interference soundscape: Many participants identified the soundscape of outdoor fitness trails as a critical factor influencing their site usage, expressing a strong preference for quiet surroundings. For instance, one participant stated: *“I prefer going in the morning when the park is less crowded; it is comparatively quiet”* (N6). Thermally comfortable microclimate: Pleasant weather and adequate shade were identified as key facilitators of outdoor exercise among older adults. Participants strongly preferred exercising in tree-shaded zones, contrasting this with the discomfort of direct solar exposure (Park B and D). As one participant commented: *“Exercising in the shade of trees is far more comfortable; I truly admire those who work out under the scorching sun without any shelter”* (N3). Equipment diversity and usability: A diverse range of equipment that met varied fitness needs was identified as a key facilitator of usage. Participants particularly favored practical, user-friendly devices.

#### 3.3.2 Environmental press factors

Adverse weather conditions: Inclement weather, such as rain and extreme heat, was identified as a major barrier to usage, as it restricted mobility and resulted in a decline in the frequency of outdoor gym use. As participants stated, *“Mainly the weather. I do not really feel like going out when it rains”* (N14). “*I do not really dare to go when it’s raining or the ground is slippery”* (N8). During extreme heat, the absence of shading in outdoor gym induced thermal discomfort, discouraging older adults from visiting. As one participant noted, *“Sometimes it’s just too hot, so I do not come. There is not much shade here, and the sun is just too intense”* (N22). Inadequate equipment: Insufficient equipment not only negatively affected older adults’ user experience but also caused long waiting times during peak hours, making it a key environmental barrier to usage. As one participant stated, *“Sometimes kids occupy the equipment, so we have to wait for a while”* (N13). *“Sometimes there are too many people here and not enough equipment, so I have to wait for a while”* (N14). Space competition and equipment occupancy issues also arose between different age groups in one case study park (Park C). Smart fitness equipment was frequently occupied by younger users, forcing older adults to resort to traditional equipment. Poor equipment maintenance: Inadequate maintenance negatively impacts older adults’ willingness to use the facilities, as aging, damage, and delayed repairs can create safety hazards and further deter usage. Noise disturbance: Excessive ambient noise negatively affected older adults’ user experience. For example, in one case study park (Park B), the children’s play area was adjacent to the senior fitness zone. One participant complained: *“Sometimes children run around, shouting, making it very noisy and disturbing our exercise”* (N17). Inadequate lighting: Poor lighting conditions (Park C and Park F) also negatively influenced older adults’ willingness to use the facilities. Participants reduced their nighttime activities due to dim lighting. One participant stated frankly: *“The lighting at night is not very good, so I feel a bit afraid of bumping into things while using the equipment”* (N29).

### 3.4 Optimization recommendations

#### 3.4.1 Safety assurance

Participants cited uneven flooring increases the risk of trips and injuries. To reduce fall risks, the floor should be leveled and finished with slip-resistant materials. Participants also called for regular maintenance and prompt repairs to ensure equipment safety and reliability. Given older adults’ reduced physical function, they recommended adding low-intensity equipment—such as low-impact cardio machines and balance trainers—to minimize exercise difficulty and injury risk.

#### 3.4.2 Information accessibility

Inadequate signage hinders wayfinding and equipment location noted that usage instructions and safety labels were too small and inconspicuous, making them hard to read during use. Participants also sought reliable exercise information, proposing that clear guidelines on proper techniques and safety tips be posted in prominent, accessible locations.

#### 3.4.3 Physical and mental comfort

The risks: participants recommended adding shade structures and rain shelters to reduce weather-related barriers. For example, one participant noted, “*I hope the park can add more shade and shelters so we can still use the equipment when the weather is bad*” (N17). Participants wanted comfortable rest areas after exercising. One respondent noted, “*I hope more benches can be added so we can rest afterward”* (N11). Additionally, storage and drinking water are needed to support basic needs and personal items during exercise.

#### 3.4.4 Appropriateness of fitness equipment

Respondents recommended expanding the variety and quantity of equipment, adding both popular machines (e.g., rowing machines, treadmills) and specialized equipment for balance and upper-body training to diversify workouts. Additionally, more low-intensity equipment is needed to accommodate users of different ages and abilities. Participants value professional support for safe exercise, but current sites lack qualified instruction and health services. Addressing this gap would strengthen social support for their physical activity.

## 4 Discussion

### 4.1 The feasibility and necessity of outdoor gym as an environmental intervention

Older adults strongly value the health benefits of outdoor gym, recognizing their positive impact on physical, mental, and social well-being. Consistent with previous studies, outdoor gym are used by older adults for fitness and recommended by clinicians for chronic disease management and rehabilitation (20, 23) maintaining health is their main motivation for using these facilities. Green exercise research have demonstrated that natural settings and physical activity produce synergistic health benefits, with greater levels of psychological restoration, including decreased stress, better emotional well-being, and enhanced self-esteem (40). This synergistic benefit was vividly exemplified by older adults exercising near the water in Park B and D, which echoes previous research attributes the mental health benefits of outdoor gym to the restorative effects of nature, suggesting it enhances these psychological gains (23). Research also shows that outdoor gym act as an “outdoor living room,” encouraging social interaction and providing vital emotional support for solitary older adults (23, 24).

Findings show that respondents actively use urban park fitness trails, with most spending over 30 min per session and exercising at least three times weekly. This contrasts with earlier studies showing that most older adults use fitness equipment for under 30 min, and that broader population averages are under 9 min (41). While this discrepancy potentially indicates that older adults with functional limitations possess a more pronounced demand for fitness, it likely reflects a self-selection bias, as older adults who agreed to participate in interviews tended to be more physically active and were therefore overrepresented in the sample. Crucially, exercising more frequently does not necessarily mean exercising correctly. Self-directed exercise is frequently accompanied by a heightened risk of physical injuries resulting from improper equipment operation (41), a concern that was corroborated by the interviewees, who explicitly suggested the integration of professional exercise guidance. Previous studies emphasize the positive role of professional instruction in facility utilization. Evidence indicates that these programs provide clear safety guidelines, lower barriers to equipment use, and consequently improve participation and physical activity levels among older adults (42). However, no older adult mentioned such courses, underscoring deficits in age-friendly design and supportive interventions for outdoor gym in China. This implies that the health benefits of outdoor gym in urban park transcend spatial efficiency, reflecting a need for individualized environmental interventions. Consequently, optimizing designs to facilitate exercise prescriptions for older adults with diverse health conditions is a key priority for age-friendly design research and practice.

Consistent with previous findings (25), this study found that strength and aerobic equipment are the most frequently used types. Although specific equipment used by older adults varies across studies, a consistent preference for strength and aerobic categories emerges. However, it is worth noting that conventional outdoor equipment that merely replicates traditional indoor gym facilities may not effectively target and rehabilitate specific functional limitations in older adults. For instance, while improving balance capabilities is critical for fall prevention, this dimension currently appears to be underserved in existing designs. This critical gap is underscored by previous research indicating that even a three-month period of outdoor fitness intervention yielded no statistically significant improvements in resistance strength, flexibility, or balance among older adults (43). Ultimately, the health benefits of a setting are generated through the interaction of individuals, activities, and the physical environment (44, 45). Which directly aligns with the core tenets of the ecological model. From this ecological perspective, health outcomes are not determined by spatial attributes alone, but by the dynamic fit between individual functional capacities and environmental demands. Future research and design practices should therefore develop target-specific environmental interventions tailored to varying health statuses, integrating appropriate equipment selection with precise recommendations for exercise frequency, intensity and duration.

### 4.2 The rehabilitation-oriented design of outdoor gym for active aging

Existing reviews have indicated that older adults use exercise facilities primarily for rehabilitation, rather than merely for preventive health purposes (28). Additionally, previous studies have explored the differences in environmental demands for outdoor gyms between older adults with mobility impairments and those living with stroke (27, 29). To the best of our knowledge, this study is the first to investigate the impact of various types of functional limitations (not just single impairment or specific illnesses) on outdoor gym utilization by older adults. This impact is most pronounced in equipment selection, as lower limb dysfunction, poor balance, and reduced flexibility limit the usability of standard fitness equipment. Therefore, equipment should be specifically designed to accommodate the diverse functional conditions of older adults. Integrating rehabilitative features into fitness equipment is key to designing age-friendly outdoor gym, enabling the broadest range of older adults to improve their health outcomes. While research on outdoor rehabilitation training facilities is still in its infancy, its importance is increasingly recognized (46–48). Nevertheless, outdoor rehabilitation training facilities are predominantly deployed in institutional settings, such as rehabilitation hospitals and long-term care facilities, while scant attention has been paid to integrating them into patients’ daily routines or community-based rehabilitation programs. The integration of outdoor rehabilitation training facilities with outdoor gym, to render fitness equipment rehabilitation-oriented and rehabilitation services routine, constitutes an indispensable pathway to achieving healthy aging. Furthermore, operating fitness equipment entails a level of technical complexity that poses challenges for older adults, making clear instructional signage is essential. Current instructional signage for fitness equipment suffers from age-unfriendly design flaws, including undersized fonts, the absence of auditory prompts, and ambiguous graphics. These features impede usage by older adults with visual or cognitive impairments.

### 4.3 Change environmental press factors to environmental support factors

Guided by the ecological model of aging, the utilization of outdoor gyms depends heavily on the dynamic balance between individual competence and environmental press. In reality, a severe conflict arises when these environmental barriers meet the functional limitations of older adults. When standard spatial configurations fail to accommodate age-related physical declines, environmental press intensifies significantly. To mitigate these psychological barriers and anxiety while fostering exercise confidence and self-efficacy, outdoor gyms must prioritize safety design. This can be achieved by providing flat, non-slip surfaces, ensuring meticulous maintenance, and selecting appropriate equipment. Indeed, empirical evidence indicates that surface material is the micro-environmental feature that most heavily influences older adults’ willingness to use outdoor fitness spaces, with rubber flooring being the most preferred option, followed by natural grass. Conversely, loose materials such as wood chips and hard surfaces such as concrete or asphalt are the least favored (29). Given that weather patterns significantly influence the occurrence of outdoor falls (49, 50), the pavement condition in outdoor gyms becomes even more critical in regions with frequent rainfall. Consistent with previous research (23, 25–27), this study finds that the selection, configuration, and maintenance of outdoor fitness equipment are critical aspects of age-friendly design. As recommended by older adults, it is essential to diversify equipment types by prioritizing low-intensity and high-demand options, along with providing clear signage and regular maintenance, to sustain their engagement.

The findings indicate that perceived comfort along outdoor gym in urban park directly impacts older adults’ willingness to use these venues. While previous studies have emphasized the importance of sun and rain shelters (26, 27, 42), this study further identifies a quiet acoustic environment as a facilitator of site use, whereas lighting and noise act as barriers to older adults’ exercise intentions. While previous literature, alongside the present study, indicates that socialization serves as a primary motivator for using outdoor gyms (23, 24), a certain level of ambient human activity and conversation can actually function as an attractor for older adults. Rather than indiscriminately pursuing absolute tranquility, designers should seek a deliberate equilibrium between mitigating physical traffic noise and preserving positive, socially vibrant sounds. Furthermore, although the significance of shade is well-established, recent evidence suggests that comfortable rest areas may exert a stronger influence on the usage willingness of older adults with mobility impairments than shade itself, primarily due to their heightened demand for immediate resting places, a preference that was also echoed by the interviewees in this study (27). Additionally, while this research demonstrates that inadequate illumination deters older adults from utilizing these spaces (as specifically emphasized by fearful respondents in Park C and Park C), prior study indicate that lighting facilities are of low importance (27). This discrepancy may stem from variations in the health statuses of the older adults sampled across different studies, as individuals with functional limitations often harbor a greater fear of falling, thereby necessitating higher visual clarity and illumination levels.

A high-quality natural environment in urban parks is a significant impact factor. Evidence indicates that exposure to natural environments elicits positive affect and reduces perceived fatigue, thereby supporting greater exercise intensity and longer durations (51, 52). It has been noted in the literature that outdoor gyms function not only as exercise spaces but also as hubs for social support and nature contact among older adults (24). Nonetheless, previous research indicates that older adults often express concerns regarding fallen leaves making the ground slippery, thereby increasing the risk of falls for individuals with functional limitations. Concerns have also been raised that excessively dense foliage might attract insects or compromise sightline openness within the fitness zone (29). Therefore, integrating these specific detailed considerations into the design phase is essential for maximizing the health benefits of the natural environment.

### 4.4 Methodological suitability and limitations

The physical and psychological experiences of older adults during equipment exercises, alongside their perceptions of spatial barriers, are inherently subjective and complex. Standardized questionnaires often fail to capture such nuanced needs, whereas a qualitative approach empowers participants to articulate their lived experiences in their own words. Furthermore, outdoor fitness behavior is highly context-dependent. Conducting on-site interviews immediately after equipment use or during direct exposure to specific spatial configurations captures real-time environment-behavior interactions, thereby minimizing retrospective recall bias. Building on these qualitative insights, future work could integrate interdisciplinary evidence to develop age-friendly design guidelines for urban park outdoor gyms, ultimately promoting active lifestyles through targeted spatial interventions. Nonetheless, several methodological limitations remain. First, regarding selection bias, the study omitted insights from individuals with severe functional impairments who were already deterred or excluded by the inadequate environment. Second, while this study categorizes relevant functional limitations and environmental factors, their precise causal mechanisms and degrees of influence on equipment exercise behavior remain unquantified. Third, regarding participant screening, individuals with severe cognitive impairments were excluded solely through verbal communication during recruitment. Future studies should employ more rigorous and standardized screening instruments, such as the Montreal Cognitive Assessment (MoCA) or the Mini-Mental State Examination (MMSE), to ensure higher methodological precision. Finally, this study adopted thematic analysis, which primarily evaluated data through existing theoretical lenses. Future research could utilize alternative qualitative approaches, such as grounded theory or ethnography, to foster bottom-up theoretical construction, thereby providing a deeper analysis of the environment-behavior interaction patterns among older adults with functional limitations in outdoor gyms.

## 5 Conclusion

The findings suggest that outdoor gym in urban park play an important role in older adults’ physical and mental health self-management. This analysis of the different needs of functional limitation older adults provides an empirical foundation for advancing the rehabilitation-oriented design of outdoor fitness equipment and promoting age-friendly transformation of outdoor gym. Furthermore, this study synthesizes the enabling and limiting environmental factors influencing older adults’ use of outdoor gyms, offering a baseline for investigating the quantitative mechanisms linking environmental characteristics to their fitness behavior. Ultimately, these findings contribute to a more nuanced understanding of environment-behavior interactions, providing a localized reference for supporting healthy aging through age-friendly design.

## Data Availability

The data analyzed in this study is subject to the following licenses/restrictions: the dataset contains anonymized information to protect individual privacy. Users are required to ensure no attempts to re-identify individuals are made. Any use that may violate ethical guidelines or privacy regulations is strictly forbidden. Requests to access these datasets should be directed to Tongyue Zhou 214249130@qq.com.

## AppendixSemi-structured interview guide

### Section 1: Motivations, perceived benefits, and usage patterns

What are your primary reasons for utilizing this outdoor gym space, and why do you choose to exercise here?

What specific benefits do you believe you gain from using this fitness equipment, considering physical, psychological, social, or economic aspects?

Could you describe in detail how you typically utilize this outdoor gym ? (e.g., how frequently you visit, at what times of day, and how long each session usually lasts).

Which types of fitness equipment do you use most frequently, and what are the reasons for your preference?

Do you engage in other exercise habits, and what distinct advantages does equipment-based exercise offer compared to those alternative activities?

### Section 2: Impacts of functional limitations on exercise behavior

You previously mentioned experiencing function limitation (such as,cognitive decline, lower limb dysfunction, visual impairment, poor cardiopulmonary endurance,auditory decline...... ). How have these functional limitations affected or altered your outdoor fitness activities?

Are you familiar with the correct operational methods for these devices, and have your functional limitations caused any specific inconveniences or safety concerns while using them?

### Section 3: Environmental press and support within the space

On a scale from 1 to 10, how would you rate this outdoor gym space, and what elements drive your satisfaction or dissatisfaction?

What specific environmental features encourage or facilitate your usage of this outdoor gym?

What specific environmental barriers deter, hinder, or exclude you from utilizing this outdoor gym?

### Section 4: Recommendations for future spatial and facility improvements

If the park management plans to upgrade and renovate this outdoor gym, what specific improvements or design interventions would you recommend?

If given the opportunity, what additional types of fitness equipment or supportive features (such as lighting, signage, or professional guidance) should be introduced?

Is there anything else regarding this urban park outdoor gym that you would like to share or elaborate on?
